# In Vitro Effects of Ligand Bias on Primate Mu Opioid Receptor Downstream Signaling

**DOI:** 10.3390/ijms21113999

**Published:** 2020-06-03

**Authors:** Xiao Zhang, Shaurita D. Hutchins, Bruce E. Blough, Eric J. Vallender

**Affiliations:** 1 Program in Neuroscience, University of Mississippi Medical Center, Jackson, MS 39216, USA; xzhang3@umc.edu; 2 Department of Psychiatry and Human Behavior, University of Mississippi Medical Center, Jackson, MS 39216, USA; shutchins2@umc.edu; 3 Center for Drug Discovery, Research Triangle Institute, Research Triangle Park, NC 27709, USA; beb@rti.org; 4 Tulane National Primate Research Center, Covington, LA 70433, USA

**Keywords:** opioid, biased agonism, morphine, nonhuman primate, second messenger signaling

## Abstract

Interest has emerged in biased agonists at the mu opioid receptor (MOR) as a possible means for maintaining potent analgesis with reduced side effect profiles. While approaches measuring in vitro biased agonism are used in the development of these compounds, their therapeutic utility will ultimately be determined by in vivo functional effects. Nonhuman primates (NHPs) are the most translational model for evaluating the behavioral effects of candidate medications, but biased signaling of these drugs at NHP MOR receptors has been unstudied. The goal of the current work was to characterize MOR ligand bias in rhesus macaques, focusing on agonists that have previously been reported to show different patterns of biased agonism in rodents and humans. Downstream signaling pathways that responded to MOR activation were identified using a luciferase reporter array. Concentration-response curves for specific pathways (cAMP, NF-ĸB, MAPK/JNK) were generated using six agonists previously reported to differ in terms of signaling bias at rodent and human MORs. Using DAMGO as a reference ligand, relative cAMP, NF-ĸB and MAPK/JNK signaling by morphine, endomorphin-1, and TRV130 were found to be comparable between species. Further, the bias patterns of across ligands for NF-ĸB and MAPK/JNK were largely similar between species. There was a high degree of concordance between rhesus macaque and human MOR receptor signaling bias for all agonists tested, further demonstrating their utility for future translational behavioral studies.

## 1. Introduction

Recent advances in the study of G-protein coupled receptor (GPCR) pharmacology have demonstrated that various intracellular pathways can be differentially activated in a ligand-specific manner, a phenomenon referred to as biased agonism [1]. Accordingly, efforts are increasing to develop drugs that selectively activate therapeutic-related pathways at GPCRs while minimizing activation of pathways associated with harmful or unwanted, but on-target, side effects, including the mu opioid receptor (MOR) [2]. Early evidence suggested that analgesia at the MOR was mediated through G-protein signaling, while side effects, particularly respiratory depression and constipation, were dependent on the recruitment of β-arrestin 2 and downstream effectors [3,4,5,6]. Given the severity of the social and financial costs of the current opioid crisis in the United States [7], developing new medications for the treatment of pain with reduced side effects is a major public health priority, and G-protein biased MOR agonists have received interest as next-generation analgesics [8,9].

Several promising studies [10,11,12,13,14] have led to the excitement that biased ligands may be an effective alternative to traditional opioids [15,16]. Other studies in rodents, however, have not shown the same reductions in side effects [17,18,19,20,21,22]. This disconnect between in vitro assays and in vivo behavior may result from numerous factors, including binding kinetic effects on signaling [23], receptor availability [17], and cellular context [24]. Species differences in the receptor may also play a role, however. Explicit comparisons of biased agonists between human and rodent kappa opioid receptors found significant species differences [25,26], as well as magnitude, if not in rank order, for ligand bias in the MOR [27].

Due to their genetic and brain morphology similarities to humans, nonhuman primates (NHPs) are the most translational preclinical model for predicting the therapeutic and toxicological effects of candidate medications, including opioid analgesics [28,29,30,31,32,33]. The same behavioral endpoints for the development of novel opioid treatments in rodents are also routinely used in NHPs: analgesia, abuse liability, tolerance and dependence, and toxicity, including respiratory depression [34,35,36]. Given the increasing attention on the development of biased MOR agonists as next-generation analgesics, an important next step will be to test these compounds in NHPs; however, there are currently no published data investigating signaling bias of agonists on NHP MOR receptors. Rhesus macaques and human MORs share 98.5% identity, differing only in 6 amino acids of 400. In comparison, the mouse and rat MOR shares 94% identity (376/400) with humans. In order to meaningfully compare in vivo behavioral data across species, it will be relevant to quantify in vitro species differences. Furthermore, a comparison of signaling bias across species is necessary to understand and validate the translation relevance of these studies.

Part of the reason why the majority of in vitro assays investigating signaling bias are done largely (or solely) in humans is because of the widespread use of assays for measuring β-arrestin recruitment based on enzyme fragment complementation technology. The commercially available tools for this approach, which requires the construction of modified fusion proteins for either β-arrestin or both GPCR and β-arrestin [37], only exist for human and limited rodent receptors. Apart from concerns that these chimeric proteins may not recapitulate physiological activity, this has limited the universe of receptors that are possible for study. One alternative method to identify ligand bias focuses not on the recruitment of β-arrestin, but rather the effects of receptor activation on downstream signaling pathways. β-arrestin serves as a scaffold protein for numerous downstream signaling pathways, including MAPK/JNK, MAPK/ERK, NF-ĸB, and others [38,39], and it is through these that the in vivo effects are presumed to be actuated [40,41]. Assays that measure the activation of these signaling pathways through luciferase reporters are commercially available and can easily be adapted to any GPCR of interest, making it well suited for the comparative studies here.

The overall goal of this study was to compare relative potencies and efficacies of a series of MOR agonists that have been reported to vary in terms of signaling bias in rhesus macaque (*Macaca mulatta*) and human MORs, focusing specifically on downstream signaling pathways as a downstream proxy of G-protein and β-arrestin recruitment. The overall hypothesis was that there would be a high degree of concordance in the bias factors for each of the compounds between rhesus macaque and human MORs.

## 2. Results

### 2.1. Downstream Signaling Pathway Responses to MOR Activation

**Figure 1:**
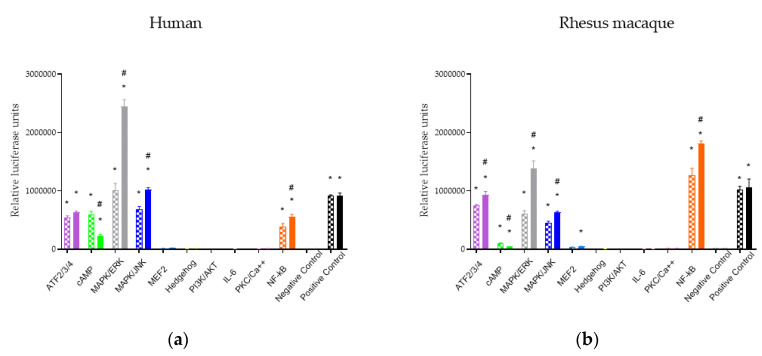
cAMP, NF-ĸB, MAPK/JNK, and MAPK/ERK respond to MOR activation by 10 µM DAMGO (solid bars) compared to saline (hatched bars) in both species, (**a**) human and (**b**) rhesus macaque. * indicates *p* < 0.05 compared to the negative control, # indicates *p* < 0.05 in the DAMGO group compared to saline.

Activation of downstream signaling pathways in cells containing MORs from both human and rhesus macaque was assessed in the presence and absence of ligands using pathway-specific luciferase reporters (Figure 1). HEK293 cell lines containing either human or rhesus macaque MORs were reverse transfected into a Cignal Finder GPCR Signaling 10-Pathway Reporter Array (Qiagen; Valencia, CA, USA) that contained inducible transcriptional factor responsive luciferase reporter constructs for common GPCR downstream signaling pathways: ATF2/3/4, cAMP, MAPK/ERK, MAPK/JNK, MEF2, Hedgehog, PI3K/AFT, IL-6, PKC/Ca^2+^, and NF-ĸB. Because MOR is known to be G_i_-linked, forskolin was added to the cells containing the cAMP reporter. ATF, cAMP, MAPK/JNK, MAPK/ERK, and NF-ĸB pathways showed significantly higher luciferase signals than the negative control under baseline treatment with saline, suggesting an endogenous activation of these pathways in the cell line or, in the case of cAMP, following forskolin addition. Downstream signaling following activation of MORs was tested using 10 μM DAMGO. Following DAMGO treatment, MAPK/JNK, MAPK/ERK, and NF-ĸB were significantly upregulated and cAMP was significantly downregulated for receptors from both species. These data demonstrate that for DAMGO, both rhesus macaque and human MORs showed similar responses on downstream pathways.

### 2.2. Effects of MOR Activation on NF-ĸB, MAPK/JNK, and cAMP Pathways Across Ligands

To understand the potency and efficacy of different ligands across downstream signaling pathways in response to activation of MORs, we focused on the subset of pathways identified above (cAMP, MAPK/JNK, and NF-ĸB). Although this study is agnostic to the mechanisms through which the signaling pathways are ultimately activated or inhibited in an effort to capture the presumed bias present in the agonists, we focused on these specific pathways to represent downstream results of both G-protein and β-arrestin response. We transduced the luciferase reporter constructs from each of the three chosen signaling pathways into HEK293 cells and created the stable cell lines through puromycin selection. To verify the selection results and confirm reporter response, we used GPCR independent substrates to stimulate each signaling pathway. Treated cells showed significantly higher luciferase readings than saline-treated cells (Figure S1), demonstrating the success of cell line generation.

**Figure 2:**
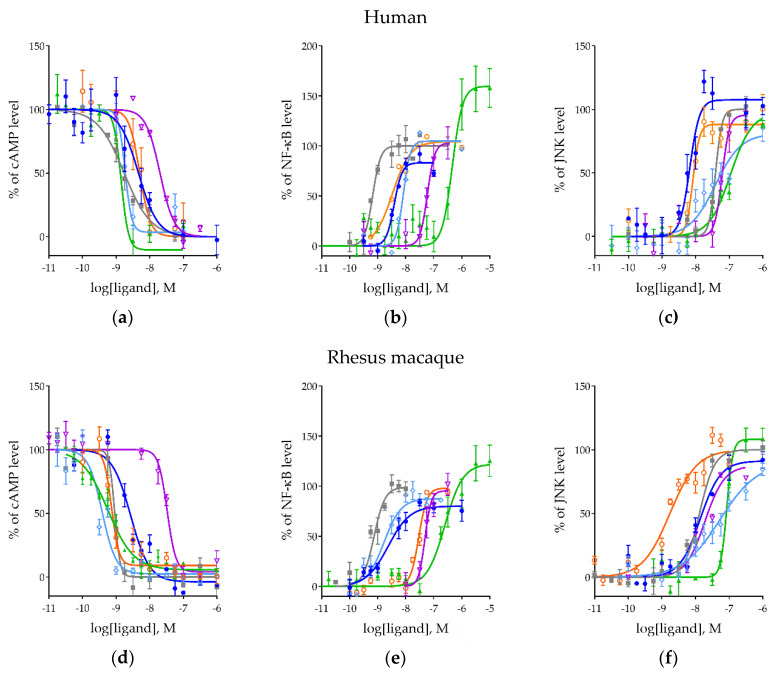
The concentration-dependent inhibition/activation of signaling by MOR ligands. Inhibition of forskolin-induced cAMP activation in human (**a**) and rhesus macaque (**d**) MORs. Stimulation of NF-ĸB in human (**b**) and rhesus macaque (**e**) MORs. Stimulation of MAPK/JNK in human (**c**) and rhesus macaque (**f**) MORs. Morphine (blue), met-enkephalin (cyan), endomorphin-1 (orange), β-endorphin (purple), and TRV130 (green).

Human and rhesus macaque MORs were then transfected into the cell lines containing the signaling reporters. These cell lines were expanded and treated with six MOR ligands: morphine, DAMGO, met-enkephalin, β-endorphin, endomorphin-1, and TRV130. The generated concentration-response curves are shown in Figure 2, and the corresponding logEC_50_, E_max_ of each curve and the species comparisons are shown in Table 1.

For the cAMP pathway, all ligands demonstrated a concentration-dependent downregulation as expected in both human and rhesus macaque MORs (Figure 2a,d). No statistically significant differences in efficacy were observed in the present experimental paradigm. For potency, the rank order in humans was similar to that observed in rhesus macaques. The slight differences in rank order between species were due to the increased potency in rhesus macaques of met-enkephalin and endomorphin-1 (Table 1). All ligands, with the exception of β-endorphin, showed consistently greater potencies at the rhesus macaque MOR than the human.

At the NF-ĸB pathway, all ligands produced a concentration-dependent stimulation in both species (Figure 2b,e), again with similar rank orders in potency with only slight differences due to met-enkephalin and endomorphin-1 (Table 1). A significant increase in potency for morphine, met-enkephalin, and TRV130, and a decrease of endomorphin-1 was observed on NF-ĸB when compared between species (Table 1). No species differences were found on E_max_ except TRV130 was significantly higher than multiple ligands in both species.

The MAPK/JNK pathway also showed a concentration-dependent activation across all ligands in both species (Figure 2c,f). The rank order of potency of ligands again tracked between species with the exception of met-enkephalin, though slight, but significant potency differences were observed for DAMGO, β–endorphin, and endomorphin-1 (Table 1). E_max_, again, showed no significant differences between species (Table 1) though there was a trend towards lower efficacy of many of the ligands compared to DAMGO.

Across all of the signaling pathways tested, we found similar potency rank orders between species. Almost the entirety of the variation that was observed resulted from differences in the endogenous ligands met-enkephalin and endomorphin-1. Each signaling pathway showed a unique rank order, however, reflecting the existence of ligand bias on these signaling pathways. Most notably, TRV130 was the most potent of the ligands tested on the cAMP pathway, but the least potent at both the NF-ĸB and MAPK/JNK pathways.

### 2.3. Ligand Bias and Species Differences Exist for MORs

**Figure 3:**
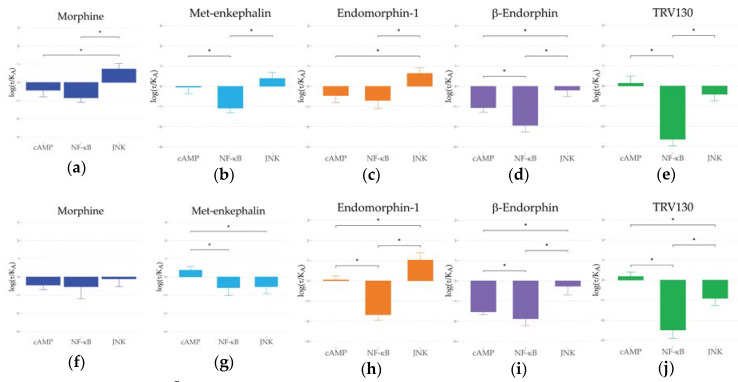
The ∆log($\frac{\tau }{K_{A}}$) values from three pathways for all ligands are shown for human (**a**–**e**) and rhesus macaque (**f**–**j**) MORs. * indicates *p* < 0.05 between signaling pathways.

The main focus of these studies was not on absolute differences in potency and efficacy between species, but rather differences in ligand bias. This allowed us to focus on patterns of downstream signaling within species and experimental systems. To do this, we assessed the bias pattern using the standard operational model of Black and Leff [42]; this takes into account both efficacy and potency, normalizing first to a standardized ligand, arbitrarily but consistently chosen to be DAMGO in MOR bias experiments, and then to a standardized pathway. This is accomplished by first calculating transduction coefficients ($\frac{\tau }{K_{A}}$) then normalizing them to the reference ligand DAMGO, as ∆log ($\frac{\tau }{K_{A}}$) (Table 2 and Figure 3).

In the human MOR, a consistent pattern emerges for morphine (Figure 3a), met-enkephalin (Figure 3b), and endomorphin-1 (Figure 3c). Each of these ligands showed a relative bias towards the MAPK/JNK pathway compared to the cAMP and NF-ĸB pathways. This general pattern was also seen in β-endorphin (Figure 3d), albeit with a small shift towards lower transduction coefficients than DAMGO across all pathways. In contrast, TRV130 (Figure 3e) showed a shift towards the cAMP pathway compared to both the MAPK/JNK and NF-ĸB pathways.

In the rhesus macaque MOR, morphine (Figure 3f) was statistically indistinguishable from DAMGO. Qualitatively, endomorphin-1 (Figure 3h), β-endorphin (Figure 3i), and TRV130 (Figure 3j) showed very similar effects on signaling on rhesus macaque MOR as they had on human MOR with differences only in degree rather than direction. Only in met-enkephalin (Figure 3g) did the rhesus macaque signaling patterns shift.

**Figure 4:**
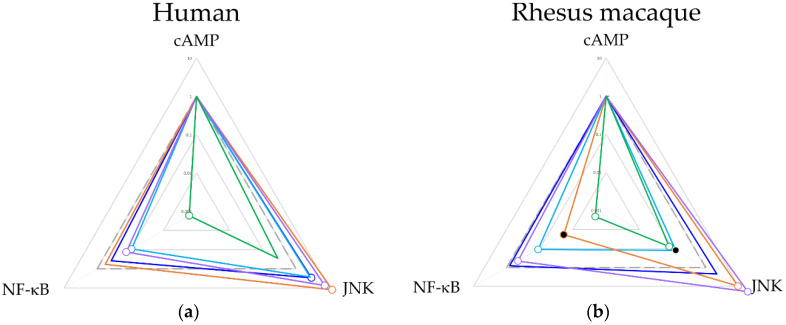
Web chart reflecting the calculated bias factor from ∆∆log($\frac{\tau }{K_{A}}$) for all ligands in human (**a**) and rhesus macaque (**b**). Empty circles represent *p* < 0.05 on ∆log($\frac{\tau }{K_{A}}$) values compared between the test ligand and the reference ligand DAMGO. Closed circles represent *p* < 0.05 on ∆log($\frac{\tau }{K_{A}}$ ) values comparisons between human and rhesus macaque. Morphine (blue), met-enkephalin (cyan), endomorphin-1 (orange), β-endorphin (purple), TRV130 (green).

Next ∆∆log ($\frac{\tau }{K_{A}}$) and bias were calculated (Table 2 and Figure 4) to determine the shifts in ligand signaling relative to cAMP inhibition. In both species, TRV130 showed a strong bias towards the cAMP pathway. Morphine showed a bias towards the MAPK/JNK pathways in humans but was statistically indistinguishable in rhesus macaques. β-endorphin and endomorphin-1 were significantly biased towards the MAPK/JNK pathway and away from the NF-ĸB pathway in both species, but the latter shift was significantly larger in rhesus macaques. For met-enkephalin, the bias away from the NF-ĸB pathway was observed in both species, but MAPK/JNK was statistically indistinguishable from DAMGO, though it was significantly lower in rhesus macaques compared to human.

Comparable trends of ∆log ($\frac{\tau }{K_{A}}$) between rhesus macaques and humans on three signaling pathways were found. NF-ĸB < cAMP < MAPK/JNK was observed for DAMGO, morphine, endomorphin-1, and β-endorphin and NF-ĸB < MAPK/JNK < cAMP was observed for TRV130 in both species. Only for met-enkephalin in rhesus macaques did the rank order of signaling pathways shift between the two. After further normalization to a common pathway (cAMP), comparable ∆∆log ($\frac{\tau }{K_{A}}$) and bias factors between species were found, although minor differences were observed. Met-enkephalin was less biased to MAPK/JNK and endomorphin-1 was less biased to NF-ĸB in rhesus macaques than in humans. Notably, morphine, β-endorphin, and TRV130 showed statistically indistinguishable patterns of bias between the species.

## 3. Discussion

This study used a luciferase-based technique to quantify signaling for a series of agonists at downstream pathways, including cAMP, NF-ĸB, and MAPK/JNK in both rhesus macaque and human MORs. The cAMP signaling pathway is commonly used to quantify G-protein coupled activity, though there are various methodological approaches to doing so. MOR is well established as a G_i_ linked GPCR; cAMP inhibition occurs after MOR activation. Here, we confirmed this downregulation of cAMP signaling by MOR activation across the studied ligands and characterized differential potencies, though not different efficacies. We observed comparable rank order of cAMP potencies across ligands between species, predicting similarities between rhesus macaque and human on behavioral and physiological effects associated with cAMP response. G-protein signaling has previously been suggested to play a crucial role in the analgesic properties of MOR ligands, and a bias towards these signaling pathways has been suggested to reflect an improved therapeutic window [27,43,44].

In this study, we very explicitly did not measure β-arrestin recruitment directly. Previous work has demonstrated that activation of MAPK/JNK is driven by β-arrestin2 recruitment [45], suggesting that measuring MAPK/JNK may be an appropriate proxy for β-arrestin2 recruitment. When we compared the present work on MAPK/JNK signaling to previous literature on β-arrestin2 recruitment using human MORs, we observed many commonalities. Previous studies indicated that in HEK293 cells, the ranking order of β-arrestin2 bias over GTPγS from low to high was DAMGO, met-enkephalin, morphine, endorphin-1 [46,47,48,49], identical to our observation for MAPK/JNK bias in human MORs. Another study in the CHO cell line also found an identical rank order of bias between GTPγS and β-arrestin2, although this did not extend to their findings for cAMP and β-arrestin2 [50]. TRV130 was developed and demonstrated as a G-protein biased ligand compared to β-arrestin2 recruitment [51]. In our study, we also showed that TRV130 demonstrated a strong cAMP bias over MAPK/JNK compared to other ligands. Together, these data are consistent with MAPK/JNK bias in our studies corresponding to β-arrestin2 bias in previous studies. Regardless, however, of whether MAPK/JNK signaling is a true proxy for β-arrestin2 recruitment, we suggest that physiologically it is the more relevant measure for interpreting behavioral outcomes.

In this study, we also characterized the effects of MOR activation and ligand biased agonism on NF-ĸB signaling. NF-ĸB has diverse roles in opioid-related behaviors, such as emotional stress and chronic opioid administration and may potentiate the expression of MORs and β-endorphin peptides [52,53,54]. Activation of NF-ĸB has previously been shown to be associated with MOR activation in response to opioid ligands, including DAMGO and morphine [55,56]. There is limited work focused on β-arrestin1 recruitment, but one study has demonstrated that endomorphin-1 and β-endorphin are biased towards to β-arrestin2 over β-arrestin1 [50]. This observation is similar to the relationship we observe between MAPK/JNK and NF-ĸB, possibly indicating a relationship between β-arrestin1 and NF-ĸB in MORs although further studies are needed. Again, however, we emphasize that the downstream signaling may be a better representation of the compound’s ultimate physiological and behavioral effects.

This study also demonstrates a largely comparable ligand biased profile between species. TRV130 and β-endorphin, in particular, showed a very similar bias pattern between species. Although morphine was found to be significantly more biased towards MAPK/JNK than DAMGO in humans but not rhesus macaques, no significant difference was observed between the species (Figure 4). Together, these findings suggest that in future in vivo studies, rhesus macaques should be expected to meaningfully model human MOR response. The only significant differences we observed were found on two endogenous ligands, met-enkephalin, and endomorphin-1. Met-enkephalin was less biased to MAPK/JNK in rhesus compared to humans, and endomorphin-1 was less biased to NF-ĸB in rhesus macaques than in humans. The causes for these differences are unclear and how these differences contribute to the behavioral differences between rhesus and human require further studies.

There are two important caveats to note in our study. First, there may be cell line-specific effects. The HEK293 cell lines used here may not be comparable to other cell lines or to neurons in vivo. However, while there may be differences between cellular contexts, there is little evidence to suggest that the differences between receptor variants systematically vary across these different contexts. Put another way, while the relative levels of signaling in the brain may differ from that in HEK293 cells, the differences and similarities between the species are expected to be similar. Similarly, receptor expression levels are common across our study, but may not recapture the physiological conditions, a criticism recently leveled at the field more generally [17]. Again, while this is a valid concern, we do not expect the differences between species to change based on this context, nevertheless, they will certainly be a fruitful area for further exploration.

These studies offer a counterpoint to more traditional in vitro studies of ligand bias, both for GPCRs generally and the MOR in particular. By focusing on effects on downstream signaling, not only is it possible to more finely understand differences in ligand consequence, but also to more carefully tie proximal molecular effects to behavioral output. Finally, this approach is more malleable to studies of receptors that deviate from standard human and rodent proteins. This allows for further investigation of other species, allelic variation, alternative splice forms, and functional mutagenesis studies, while still affording tests of activity and ligand bias.

We evaluated the downstream signaling patterns evoked by human and rhesus macaque MOR activation using luciferase reporter techniques. Overall, the tested ligands demonstrate bias at signaling pathways with the MAPK/JNK bias of studied ligands comparable to previous studies on β-arrestin2 bias. We also found largely comparable signaling bias profiles between humans and rhesus macaques. These studies can inform behavioral and physiological organismal studies in nonhuman primates using MOR ligands and support translational validity during the development of novel biased ligands as therapeutic ligands.

## 4. Materials and Methods

### 4.1. Plasmids

pcDNA3.1/V5-His-TOPO vectors (Life Technologies; Grand Island, NY, USA) containing either human (NM_000914) or rhesus macaque (NM_001032824) *OPRM1* were generated following previously described methods [57]. Cloned *OPRM1* plasmids were transformed into DH5-α competent cells (Invitrogen; Waltham, MA, USA) following the manufacturer’s protocol, and bacteria were shaken at 37 °C at 225 rpm overnight in LB medium (Invitrogen). Plasmids were purified using the Plasmid Plus Maxi kit (Qiagen; Valencia, CA, USA), confirmed by Sanger sequencing, and stored at −20 °C until use.

### 4.2. Cell Lines

HEK293 cell lines were purchased (ATCC; Manassas, VA, USA) and maintained in Dulbecco’s modified Eagle’s medium (DMEM) supplemented with 10% FBS and 1% penicillin-streptomycin (Gibco; Waltham, MA) in 100 mm^2^ culture dishes (Corning; Corning, NY, USA) at 37 °C with 5% CO_2_. They were stably transfected with either human or rhesus macaque *OPRM1* plasmids using Lipofectamine 3000 reagent (Invitrogen) followed by selection using geneticin (Gibco) for two weeks. PCR was used to confirm gene expression.

Separately, HEK293 cell lines expressing luciferase reporters for second messenger signaling pathways were generated using stable transduction of Cignal Lenti-Reporter assays (Qiagen). These assays make use of lentiviral particles carrying luciferase reporter genes under the transcriptional control of specific second messenger signaling pathway transcriptional response elements. Here, cell lines were generated for reporting activation of the cAMP, NF-ĸB and MAPK/JNK pathways using lentiviral particles with a multiplicity of infection equal to 2. After transduction over three days, puromycin selection was performed, and cells were grown to confluence as above. Confirmation of activity was accomplished with GPCR independent substrates (Figure S1).

### 4.3. GPCR Signaling Reporter Array

To broadly survey the signaling effects following MOR activation, we used the Cignal Finder GPCR Signaling 10-Pathway Reporter Array (Qiagen). The reporter array contains inducible transcriptional factor responsive firefly luciferase reporter constructs for ten unique pathways as well as a non-inducible reporter construct as negative control and a construct that constitutively expresses luciferase and GFP as a positive control. Each inducible pathway reporter construct encodes the firefly luciferase gene under the control of a basal promoter element and tandem repeats of a transcriptional response element specific to the pathway of interest. We reverse transfected the reporter constructs into HEK293 cells expressing either human or rhesus macaque *OPRM1* using Attractene (Qiagen). The cells were then treated with either saline or the MOR agonist DAMGO (10 μM) for 6 h. Because MOR is known to be G_i_-linked [58], 1 μm forskolin was added prior to DAMGO treatment to measure inhibition of cAMP activation. We then lysed the cells and measured luciferase signals for all pathways using the Luciferase Assay System (Promega; Madison, WI, USA) on a Victor X5 plate reader (PerkinElmer; Waltham, MA, USA) as described previously [57].

### 4.4. Drugs

We chose six MOR agonists that include endogenous and exogenous ligands and that vary in terms of G-protein bias: morphine (Sigma–Aldrich; St. Louis, MO, USA), DAMGO ([D-Ala^2^-MePhe^4^-Gly-ol]-enkephalin; Bachem, Torrance, CA, USA), met-enkephalin (Sigma–Aldrich), β-endorphin (American Peptide Co., Sunnyvale, CA, USA), endomorphin-1 (Tocris Bioscience, Minneapolis, MN, USA), and TRV130 (oliceridine; synthesized by B.E.B.). All ligands except TRV130, which was first dissolved in DMSO (20%), were dissolved in water, then prepared as serial dilutions to concentrations between 10^−11^ to 10^−5^ M. GPCR independent substrates including forskolin, TNF-α and PMA (Sigma–Aldrich) were used to activate cAMP, NF-ĸB and MAPK/JNK respectively. All GPCR independent substrates were diluted in DMSO to final concentrations of 10 μM for forskolin, 50 ng/mL for TNF-α, and 10 ng/mL for PMA.

### 4.5. Concentration Response Determinations

To generate concentration-response curves, HEK293 cells stably expressing either cAMP, NF-ĸB, or MAPK/JNK luciferase reporters were first seeded in 24-well plates (Corning) overnight to 60–70% confluence. We then transiently transfected the plasmids containing either the rhesus macaque or human *OPRM1* gene into the cells using Attractene. A vector constitutively expressing GFP on the same plasmid backbone was used as a control for transfection efficiency. 48 h after transfection, ligands across a range of concentrations were added to the cells and incubated for 20 h. Cells were lysed, and the luciferase signal was measured as above.

### 4.6. Data Analysis

To verify that the luciferase reporter genes were working in our generated stable cell lines, we treated these cells with saline and substrates that stimulate the signaling pathways independent of GPCR activation (i.e., forskolin, PMA, and TNF-α) for 20 h. We then lysed the cells and measured luciferase activities. An unpaired t-test (two-tailed) was used to compare between groups, *p* value less than 0.05 was considered statistically significant following Bonferroni correction for multiple testing.

For generating the concentration-response curves, luciferase data from each condition were fit into three-parameter non-linear response curves using Prism 8.4.2 (GraphPad; La Jolla, CA, USA). An extra sum-of-squares F test was used to compare the differences of Log EC_50_ (potency) and E_max_ (efficacy) for the signaling pathways between species and between different ligands within species. Comparisons with *p* value less than 0.05 were considered to be significantly different, again using a Bonferroni correction. The E_max_ values used in this study were normalized as follows: For the cAMP pathway, cells treated with 1 μM forskolin defined 100%, and cells treated with 1 μM forskolin and 1 μM DAMGO defined 0%. For the NF-ĸB and MAPK/JNK pathways, data from saline-treated cells defined 0% and 1 μM DAMGO-treated cells defined 100%. The EC_50_ for each ligand was defined by the molar concentration required to generate a half-maximal response. To calculate bias factors, curves were fit with an operational model [42]:

$$Response=\frac{E_{m}{\left(\frac{\tau }{K_{A}}\right)}^{n}{\left[A\right]}^{n}}{{\left(\frac{\tau }{K_{A}}\right)}^{n}{\left[A\right]}^{n}+\left(1+\frac{{\left[A\right]}^{n}}{K_{A}}\right)}$$

where *E_m_* was the possible maximum response, n was the transducer slope; both parameters were equal for each calculation. A was the molar concentration of the ligand, K*_A_* was the equilibrium dissociation constant of ligand A and τ was the agonist efficacy. τ and K*_A_* were determined in the operational model, and log ($\frac{\tau }{K_{A}}$) was calculated for each concentration-response function.

Using DAMGO as the reference ligand, normalized transduction coefficients ∆log ($\frac{\tau }{K_{A}}$) was calculated by the following equation:

$$∆\log\;(\frac{\tau }{K_{A}})=\log\;(\frac{\tau }{K_{A}}){\;}_{\mathrm{test}\;\mathrm{ligand}}-\;\log\;(\frac{\tau }{K_{A}}){\;}_{\mathrm{DAMGO}}$$

Using cAMP as a reference signaling pathway to generate the double normalized transduction coefficients ∆∆log ($\frac{\tau }{K_{A}}$) followed:

$$∆∆\log\;(\frac{\tau }{K_{A}})=∆\log\;(\frac{\tau }{K_{A}}{)}_{\mathrm{pathway}}-\;∆\log\;(\frac{\tau }{K_{A}}{)}_{\mathrm{cAMP}}$$

Finally, the bias factor for each condition was calculated using the following equation:

$$\mathrm{Bias}=10^{∆∆\log\;}(\frac{\tau }{K_{A}})$$

∆log ($\frac{\tau }{K_{A}}$) and ∆∆log ($\frac{\tau }{K_{A}}$) were expressed as the mean value ± SEM, which were calculated following the formula:

$$\mathrm{SEM}=\sqrt{{\left(SEM_{pathway1}\right)}^{2}+{\left(SEM_{pathway2}\right)}^{2}}$$

For the comparisons between these parameters, we used a two-way unpaired Student *t*-test, *p* value less than 0.05 was considered statistically significant.

## Figures and Tables

**Table 1:** Summary of ligand potencies and efficacies in human and rhesus macaque MORs.

Ligand	Human		Rhesus Macaque	
	LogEC50	Emax	LogEC50	Emax
**cAMP**				
DAMGO	−8.76 ± 0.07	100.0 ± 4.09	**−9.04 ± 0.03**	100.2 ± 6.06
morphine	−8.33 ± 0.13	100.0 ± 5.35	−8.57 ± 0.08	100.9 ± 7.18
met-enkephalin	−8.74 ± 0.07	100.4 ± 14.99	**−9.42 ± 0.07**	100.0 ± 5.09
endomorphin-1	−8.30 ± 0.10	100.4 ± 12.14	**−9.14 ± 0.05**	99.6 ± 6.72
β-endorphin	−7.70 ± 0.05	100.1 ± 3.70	**−7.50 ± 0.03**	104.2 ± 1.89
TRV130	−8.85 ± 0.12	100.1 ± 6.90	**−9.25 ± 0.07**	100.0 ± 5.34
**NF-ĸB**				
DAMGO	−9.18 ± 0.07	100.0 ± 5.22	−9.23 ± 0.09	100.0 ± 7.72
morphine	−8.41 ± 0.05	83.2 ± 4.73	**−8.72 ± 0.26**	93.5 ± 11.34
met-enkephalin	−8.08 ± 0.04	104.8 ± 5.06	−8.67 ± 0.16	93.7 ± 4.68
endomorphin-1	−8.46 ± 0.15	104.2 ± 6.43	**−7.54 ± 0.04**	104.2 ± 5.21
β-endorphin	−7.23 ± 0.07	103.0 ± 12.87	−7.33 ± 0.07	103.0 ± 10.71
TRV130	−6.34 ± 0.10	159.6 ± 12.61	**−6.61 ± 0.13**	132.7 ± 11.57
**MAPK/JNK**				
DAMGO	−7.40 ± 0.08	101.8 ± 8.47	**−7.88 ± 0.10**	112.9 ± 10.59
morphine	−8.18 ± 0.07	90.8 ± 4.59	−7.86 ± 0.12	91.4 ± 7.10
met-enkephalin	−7.93 ± 0.08	72.0 ± 4.58	−7.53 ± 0.09	73.5 ± 5.33
endomorphin-1	−8.08 ± 0.06	90.9 ± 3.84	**−8.99 ± 0.09**	92.5 ± 3.49
β-endorphin	−7.20 ± 0.07	102.0 ± 8.43	**−7.71 ± 0.12**	88.5 ± 7.70
TRV130	−6.99 ± 0.07	101.3 ± 10.05	−6.93 ± 0.08	123.2 ± 8.65

Bold indicates *p* < 0.05 between rhesus macaques and human.

**Table 2:** Summary of the transduction coefficients and bias factors in MORs of human and rhesus macaques: Transduction coefficients log($\frac{\tau }{K_{A}}$), normalized (to DAMGO) transduction coefficients ∆log($\frac{\tau }{K_{A}}$), and double normalized (to DAMGO and cAMP) transduction coefficients ∆∆log($\frac{\tau }{K_{A}}$).

Ligand	Human			Rhesus Macaque		
	$\log(\frac{\tau }{K_{A}})$	$∆\log(\frac{\tau }{K_{A}})$	$∆∆\log(\frac{\tau }{K_{A}})$	$\log(\frac{\tau }{K_{A}})$	$∆\log(\frac{\tau }{K_{A}})$	$∆∆\log(\frac{\tau }{K_{A}})$
**cAMP**						
DAMGO	10.76 ± 0.18	0.00 ± 0.25	0.00 ± 0.36	11.04 ± 0.12	0.00 ± 0.17	0.00 ± 0.24
morphine	10.33 ± 0.31	−0.43 ± 0.36	0.00 ± 0.51	10.59 ± 0.21	−0.45 ± 0.25	0.00 ± 0.35
met-enkephalin	10.72 ± 0.26	−0.04 ± 0.32	0.00 ± 0.45	11.41 ± 0.18	0.37 ± 0.21	0.00 ± 0.30
endomorphin-1	10.30 ± 0.30	−0.46 ± 0.35	0.00 ± 0.49	11.09 ± 0.15	0.05 ± 0.19	0.00 ± 0.27
β-endorphin	9.70 ± 0.13	**−1.06 ± 0.22**	0.00 ± 0.32	9.50 ± 0.07	*−1.54 ± 0.14*	0.00 ± 0.20
TRV130	10.90 ± 0.30	0.14 ± 0.35	0.00 ± 0.49	11.23 ± 0.19	0.18 ± 0.22	0.00 ± 0.32
**NF-ĸB**						
DAMGO	11.18 ± 0.19	0.00±0.26	0.00 ± 0.37	11.23 ± 0.24	0.00 ± 0.34	0.00 ± 0.38
morphine	10.33 ± 0.15	**−0.85 ± 0.24**	−0.42 ± 0.43	10.69 ± 0.62	−0.54 ± 0.66	−0.09 ± 0.71
met-enkephalin	10.10 ± 0.11	−1.08 ± 0.22*	**−1.04 ± 0.39**	10.64 ± 0.36	−0.59 ± 0.43	**−0.95 ± 0.48**
endomorphin-1	10.48 ± 0.36	−0.70 ± 0.41	−0.24 ± 0.53	9.55 ± 0.11	*−1.68 ± 0.27*	*−1.73 ± 0.33*
β-endorphin	9.24 ± 0.25	**−1.94 ± 0.32**	**−0.88 ± 0.39**	9.34 ± 0.23	**−1.88 ± 0.34**	**−0.34 ± 0.37**
TRV130	8.54 ± 0.26	**−2.64 ± 0.32**	**−2.78 ± 0.48**	8.74 ± 0.34	**−2.49 ± 0.41**	**−2.67 ± 0.47**
**MAPK/JNK**						
DAMGO	9.41 ± 0.23	0.00 ± 0.32	0.00 ± 0.41	9.93 ± 0.29	0.00 ± 0.41	0.00 ± 0.44
morphine	10.14 ± 0.19	**0.74 ± 0.29**	**1.16 ± 0.46**	9.82 ± 0.31	*−0.11 ± 0.42*	0.34 ± 0.49
met-enkephalin	9.79 ± 0.22	0.39 ± 0.31	0.42 ± 0.45	9.39 ± 0.25	*−0.54 ± 0.38*	*−0.90 ± 0.44*
endomorphin-1	10.04 ± 0.17	0.64 ± 0.28	**1.10 ± 0.45**	10.96 ± 0.22	**1.03 ± 0.36**	**0.98 ± 0.41**
β-endorphin	9.21 ± 0.20	−0.19 ± 0.31	**0.87 ± 0.38**	9.66 ± 0.32	−0.27 ± 0.43	**1.27 ± 0.45**
TRV130	8.99 ± 0.22	−0.41 ± 0.32	−0.55 ± 0.47	9.02 ± 0.22	**−0.91 ± 0.36**	**−1.10 ± 0.42**

The data are shown with mean ± S.E.M. Bold indicates *p* < 0.05 versus DAMGO. Italics indicates *p* < 0.05 between species.

## Supplementary Materials

Supplementary materials can be found at https://www.mdpi.com/1422-0067/21/11/3999/s1.

## Abbreviations

MOR	µ opioid receptor
NHP	nonhuman primate
GPCR	G-protein coupled receptor
cAMP	cyclic adenosine 5′-monophosphate
NF-ĸB	nuclear factor kappa-light-chain-enhancer of activated B cells
MAPK	mitogen-activated protein kinase
JNK	Jun N-terminal kinase
ERK	extracellular-signal-regulated kinase
ATF	activating transcription factor
DAMGO	[D-Ala^2^-MePhe^4^-Gly-ol]-enkephalin
PMA	phorbol 12-myristate 13-acetate
TNF- α	tumor necrosis factor α
GFP	green fluorescent protein
