# Supplementary material for: In Vitro Effects of Ligand Bias on Primate Mu Opioid Receptor Downstream Signaling
Source: Int J Mol Sci. 2020 Jun 3;21(11):3999. doi: 10.3390/ijms21113999 (PMC7312292; doi:10.3390/ijms21113999)
Supplement: Supplementary file 1 [file ijms-21-03999-s001.pdf]

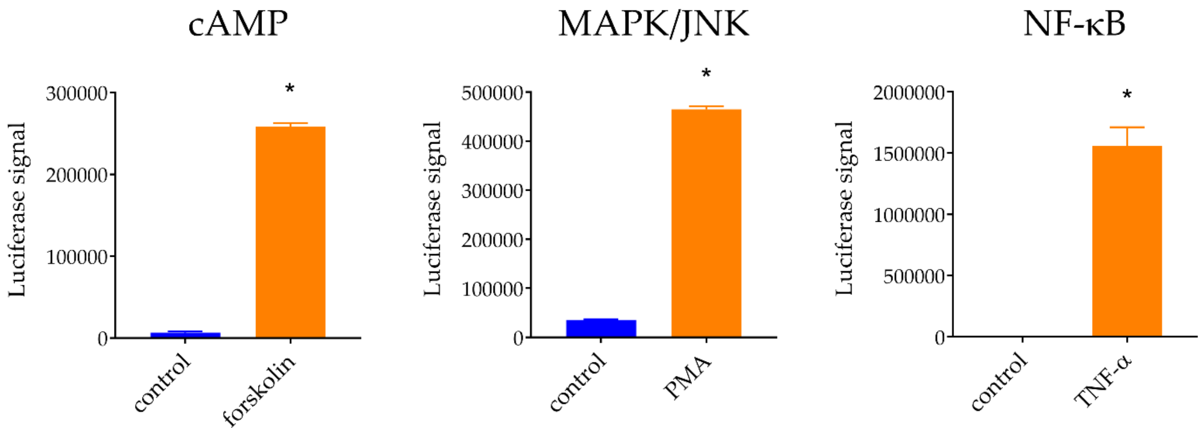

(**a**) (**b**) (**c**)

Figure S1. Comparisons of the luciferase signals on HEK293 cells transduced with (**a**) cAMP, (**b**) MAPK/JNK, (**c**) NF-κB when treated with saline ("control") or corresponding non-GPCR second messenger signal stimulants. * indicates $p < 0.05$ compared to the control.
